# Association between cardiovascular diseases and COVID-19 pneumonia outcome in Indonesia: a multi-center cohort study

**DOI:** 10.3389/fmed.2023.1190148

**Published:** 2023-06-29

**Authors:** Erlina Burhan, Farhan Mubarak, Siti Aliyah Said Utriyani Adilah, Cut Yulia Indah Sari, Efriadi Ismail, Puji Astuti, Yasmina Hanifah, Elvan Wiyarta, Nana Maya Suryana

**Affiliations:** ^1^Department of Pulmonology and Respiratory Medicine, Persahabatan Central General Hospital, Universitas Indonesia, Jakarta, Indonesia; ^2^Department of Pulmonology and Respiratory Medicine, Faculty of Medicine, Universitas Indonesia, Jakarta, Indonesia; ^3^Department of Pulmonology, Jakarta Islam Hospital Cempaka Putih, Jakarta, Indonesia; ^4^Department of Pulmonology, Yarsi Hospital, Jakarta, Indonesia; ^5^Department of Pulmonology, Cengkareng District General Hospital, Jakarta, Indonesia; ^6^Department of Cardiology and Vascular Medicine, Persahabatan Central General Hospital, Jakarta, Indonesia; ^7^Department of Medical Science, Faculty of Medicine, Universitas Indonesia, Jakarta, Indonesia

**Keywords:** prognosis, predictive, preventive care, comorbidities, length of stay

## Abstract

**Background:**

COVID-19 is a pandemic affecting 185 countries, including Indonesia. Cardiovascular diseases (CVD) in COVID-19 patients were linked to worse clinical outcomes. However, the association remained inconclusive due to limited data in Indonesia. This study aimed to determine the association between CVD in COVID-19 pneumonia patients with its clinical outcomes.

**Methods:**

This retrospective cohort study was conducted in four Indonesian hospitals, enrolling 584 adult COVID-19 pneumonia patients from September 2020 to July 2021. Patients were categorized into two groups: non-CVD and CVD [hypertension, coronary artery disease (CAD), chronic heart failure (CHF), hypertensive heart disease (HHD), arrhythmia, cardiomegaly, left ventricular hypertrophy (LVH), mitral regurgitation (MR), and myocardial injury (MI)]. Clinical outcomes include in-hospital mortality, intensive care unit admission, ventilator use, earlier death, and prolonged hospital stay. Mann–Whitney test was used for analysis.

**Results:**

The most common CVD was hypertension (48.1%), followed by MI (10.6%), CAD (9.2%), CHF (6.8%), HHD (3.1%), arrhythmia (1.7%), and others (0.7%). The in-hospital mortality rate was 24%, and patients were hospitalized for a median of 12 days. MI was the only CVD that increased in-hospital mortality (RR 2.105). It was also significantly increased in patients with diabetes mellitus (RR 1.475) and chronic kidney disease (RR 2.079). Meanwhile, prolonged hospital stay was associated with any CVD (RR 1.553), hypertension (RR 1.511), MI (RR 1.969), CHF (RR 1.595), diabetes mellitus (RR 1.359), and cerebrovascular disease (RR 2.203).

**Conclusion:**

COVID-19 pneumonia in patients with CVD, specifically MI and hypertension, worsens the COVID-19 clinical outcomes.

## Introduction

Coronavirus disease 2019 (COVID-19) has spread around the globe at an alarming rate since it was first recognized in December 2019, leading to more than 600 million confirmed cases, including over six million deaths, as of 2 September 2022 (1). The death caused by COVID-19 in Indonesia is even more frequent than in the global situation, with a mortality rate of approximately 2% (1).

Since the pandemic broke out, massive research has been conducted to identify and reveal the association between fatal or other poor clinical outcomes and pre-existing disease characteristics of patients with COVID-19 (2). Besides respiratory tract involvement, cardiovascular manifestation induced by severe acute respiratory syndrome coronavirus (SARS-CoV-2) infection prompted substantial concerns (2). In a retrospective and observational study conducted in a hospital in Wuhan, China, the mortality rate was nearly 70% in hospitalized patients with COVID-19 who had underlying cardiovascular disease (CVD), simultaneously presenting with elevated troponin T levels (2). Similar results of fatal outcomes were also reported for patients with pre-existing cardiovascular disease (CVD) in another retrospective analysis performed in a hospital in Wuhan. In addition, complications in such patients were much more common than in those without CVD, including but not limited to acute respiratory distress syndrome, acute kidney injury, and electrolyte disturbances (3). Combining clinical observations with the virus infection mechanism, characterized by direct and indirect myocardial injury (MI) and acute systemic inflammation (especially for severe COVID-19), pre-existing CVD and cardiovascular risk factors are related to heightened vulnerability to COVID-19. Conversely, acute MI and chronic damage to the cardiovascular system caused by COVID-19 can deteriorate pre-existing CVD or bring other new complications (4–6).

COVID-19’s proliferation has also had a greater impact on densely populated developing nations. Indonesia is one such country. In Indonesia, 3,666,031 COVID-19 cases have been confirmed since March 2020, as of August 8, 2021 (7). This included 474,233 (12.9%) active cases, 107,096 (2.9%) fatalities, and 3,084,701 (84.1%) recovered cases (7). In accordance with the Presidential Decree of the Republic of Indonesia regulating the determination of man-made catastrophes, Indonesia declared a national catastrophe on April 13, 2020, due to the outbreak of COVID-19 (7). Since then, the government of Indonesia has taken measures to contain the COVID-19 epidemic (7). Nonetheless, Indonesia’s epidemiological statistics currently lack information, especially on CVD. This dataset is certainly very important, especially for developing strategic policies related to COVID-19 and CVD.

Apart from Indonesia, several countries already have datasets related to the relationship between COVID-19 and CVD. Two comprehensive and systematic meta-analyses with a broad review were performed to re-examine the association between cardiac injury and outcomes in patients with COVID-19 in China. A total of 3,175 and 4,189 patients were included in the two studies, respectively (8, 9). The results were consistent with the studies conducted within a limited range (e.g., a single center), portraying a clear association between cardiac injury and a higher risk of mortality and indicating the necessity of closely monitoring heart health and utilizing effective and targeted treatment strategies (8, 9). Meanwhile, between March 2020 and June 2021 in the United States, a total of 600,241 COVID-19-related fatalities were reported (10). The most prevalent cardiovascular conditions among COVID-19-related fatalities were hypertensive diseases, diabetes, and ischemic heart disease (10). Other countries, such as Italy, also have COVID-19 datasets for CVD that reveal hypertensive disease to be the most prevalent comorbidity (11). Even in Qatar, the developed dataset was able to demonstrate that pre-existing CVD, age, and other comorbidities predict the risk of hospitalization and additional complications among COVID-19 patients (12).

The association between CVD and COVID-19 remained equivocal in Indonesia due to the limited data obtained, as compared to various datasets from other countries. Therefore, the present study aims to determine the association between CVD in patients with COVID-19 and clinical outcomes, adding value to clinical practice recommendations for local healthcare providers in Indonesia.

## Methods

### Study design and sample characteristics

This retrospective cohort multi-center study in patients with COVID-19 was commenced following approval by Independent Ethics Committee from four study centers in Jakarta, Indonesia, which included Persahabatan Central General Hospital, Jakarta Islam Hospital Cempaka Putih, Yarsi Hospital, and Cengkareng District General Hospital. The study was conducted in adherence to the principles of the Declaration of Helsinki (13).

Using consecutive sampling, adult COVID-19 pneumonia patients (moderate, severe, and critical diseases) were enrolled in the study from September 2020 to February 2021. The sample size was calculated using a proposed relative risk (RR) of 1.5, alpha 5%, and power 80%. The minimum sample size for each group was 103 patients, including 10% dropouts.

This study included adult patients (aged 18 years or older) who were suspected or confirmed COVID-19 as evidenced by the results of the RT-PCR SARS-CoV-2, hospitalized at Persahabatan Hospital, Jakarta Islamic Hospital Cempaka Putih, Yarsi Hospital, or Cengkareng District General Hospital with moderate, severe or critically case degree according to the severity classification of COVID-19 in the COVID-19 Management Guidelines in Indonesia. We admitted patients who received intravenous antiviral agents.

This study excluded patients who received double antiviral agents simultaneously, patients who enrolled in another clinical trial, patients who received therapy from another hospital, and those who were referred from or to another hospital. We also excluded patients who left the hospital within 24 h of hospitalization, including death-on-arrival patients. Since not every study hospital routinely checked troponin blood level to confirm the cause of death, we excluded patients who died within 24 h of hospitalization.

### Data collection

Electronic medical records for individual patients were collectively retrieved from the Medical Records Office and independently reviewed by different investigators. Recorded patient data included demographic characteristics, medical history, symptoms, comorbidities, complications, treatment measures, laboratory findings, and outcomes (in-hospital mortality, ICU admission, ventilator use, earlier death, and prolonged hospital stay). Earlier death was defined as death during less than 13 days of hospitalization due to a recommendation from the COVID-19 national guideline in Indonesia to hospitalize patients for at least 13 days. A prolonged hospital stay was defined as more than 13 days of hospitalization.

All patients enrolled were categorized into two groups according to the presence or absence of CVD, which was defined as any of the following comorbidities: hypertension, coronary artery disease (CAD), chronic heart failure (CHF), hypertensive heart disease (HHD), arrhythmia, cardiomegaly, left ventricular hypertrophy (LVH), mitral regurgitation (MR), and myocardial injury (MI). The medical doctor in charge diagnosed all the CVD. The history of MI, electrocardiography interpretation of old MI, or any history of percutaneous coronary intervention or bypass surgery defined CAD. MI was diagnosed by an acute chief complaint of chest discomfort, confirmed by electrocardiography interpretation of ST-wave elevation or troponin level elevation. CHF and MR was diagnosed using echocardiography.

### Statistical analysis

Before analysis, Microsoft Excel was used to input data gathering into a main table (Microsoft Corp, Redmond, WA, United States). Statistical Package for the Social Sciences (IBM Corp, Armonk, NY, United States) version 24 was used to analyze and display the tabulated data. Categorical variables were presented with frequency (percentage), and continuous variables were summarized by median (interquartile range). Bivariate analysis was calculated using the chi-square test or Mann–Whitney test. *p*-values less than 0.05 are considered statistically significant.

## Results

**Figure 1:**
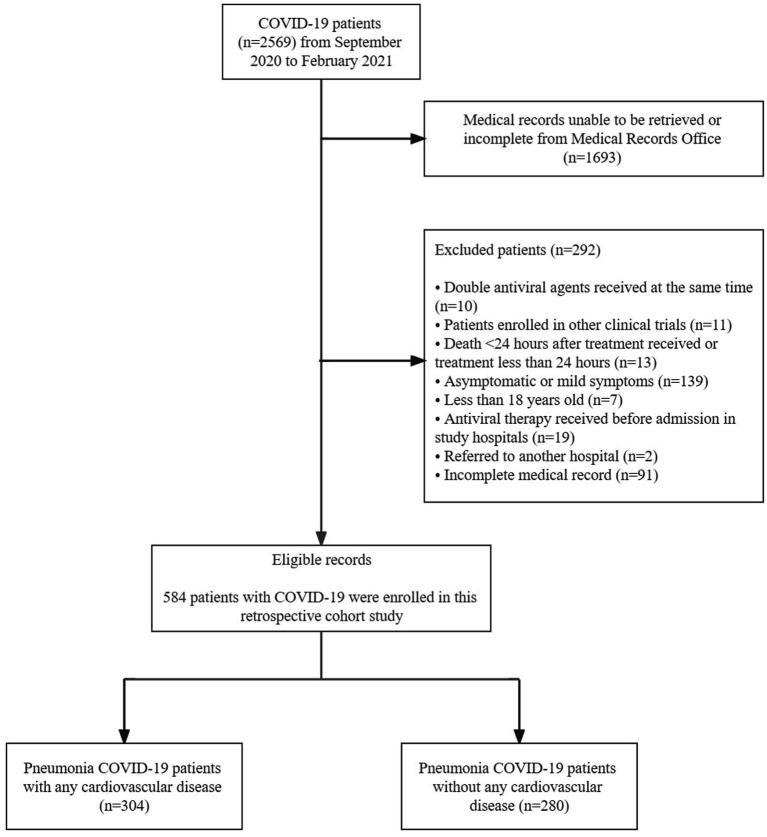
Study patients disposition.

There were 2,569 COVID-19 pneumonia patients assessed from the registries of 4 study centers, shown in Figure 1. At the end of the enrollment, 584 COVID-19 pneumonia patients were included in the study (304 patients were diagnosed with CVD, and 280 patients were without CVD). The demographic characteristics are shown in Table 1. The age group was dominated by adults aged 18–59 (64.6%) and males (59.4%). There were 4.45% of female patients who were pregnant at admission. More than half of the patients (53.8%) had mild–moderate severity of COVID-19. Among the subjects, dyspnea is the most common symptom (78.25) but did not show any significant association with cardiovascular comorbidities in COVID-19 pneumonia patients. Sore throat is significantly associated with CVD in COVID-19 pneumonia patients (*p* = 0.04). The other symptoms were also analyzed: fever, cough, nasal congestion, malaise, dyspnea, GI complaint, anosmia, ageusia, loss of consciousness, and angina, with no significant associations found.

**Table 1:** Demographics and clinical characteristics.

Characteristic	Pneumonia COVID-19 patients with		Total	*p*-value^a^	RR
	CVD	Non-CVD			
*Demographics*					
Age					
Geriatric (≥60 years old)	134 (64.7%)	73 (35.3%)	207 (35.44%)	**<0.001** ^*^	1.434
Adult (18–59 years old)	170 (45.1%)	207 (54.9%)	377 (64.56%)		
Sex					
Male	188 (54.2%)	159 (45.8%)	347 (59.4%)	0.214	1.107
Female	116 (48.9%)	121 (51.1%)	237 (40.6%)		
Pregnancy					
Pregnant	1 (3.8%)	25 (96.2%)	26 (4.45%)	**0.005** ^*^	0.07
Not pregnant	303 (54.3%)	255 (45.7%)	558 (95.55%)		
COVID-19 severity					
Mild-moderate	168 (53.5%)	146 (46.5%)	314 (53.76%)	0.45	0.941
Severe-critical	116 (50.4%)	134 (49.6%)	250 (42.8%)		
Missing			20 (3.42%)		
*Clinical presentation at admission*					
Fever					
Yes	168 (53.5%)	146 (46.5%)	314 (53.76%)	0.341	1.085
No	116 (50.4%)	96 (50.8%)	212 (36.3%)		
Missing			58 (9.93%)		
Cough					
Yes	168 (53.5%)	146 (46.5%)	314 (53.76%)	0.341	1.074
No	116 (50.4%)	96 (50.8%)	212 (36.3%)		
Missing			58 (9.93%)		
Sore throat					
Yes	20 (38.5%)	32 (61.5%)	52 (8.9%)	**0.04** ^*^	0.72
No	284 (53.4%)	248 (46.6%)	532 (91.1%)		
Nasal congestion					
Yes	15 (50%)	15 (50%)	30 (5.13%)	0.814	0.95
No	289 (52.2%)	265 (47.8%)	554 (94.87%)		
Malaise					
Yes	99 (56.6%)	76 (43.4%)	175 (29.96%)	0.153	1.128
No	205 (50.1%)	204 (49.9%)	409 (70.04%)		
Dyspnea					
Yes	240 (52.5%)	217 (47.5%)	457 (78.25%)	0.672	1.041
No	64 (50.4%)	63 (49.6%)	127 (21.75%)		
Any GI complaint					
Yes	96 (49%)	100 (47.9%)	196 (33.56%)	0.29	0.913
No	208 (53.6%)	180 (46.4%)	388 (66.44%)		
Anosmia					
Yes	16 (39%)	25 (61%)	41 (7.02%)	0.083	0.735
No	288 (53%)	255 (47%)	543 (92.98%)		
Ageusia					
Yes	1 (25%)	3 (75%)	4 (0.68%)	0.277	0.478
No	303 (52.2%)	277 (47.8%)	580 (99.32%)		
Loss of consciousness					
Yes	5 (71.4%)	2 (28.6%)	7 (1.19%)	0.302	1.379
No	299 (51.2%)	278 (48.2%)	577 (98.81%)		
Angina					
Yes	3 (37.5%)	5 (62.5%)	8 (1.37%)	0.407	0.717
No	301 (52.3%)	275 (47.7%)	576 (98.63%)		
*Comorbidities*					
Diabetes mellitus					
Yes	161 (66%)	83 (34%)	244 (41.78%)	**0.005** ^*^	1.569
No	143 (42.1%)	197 (57.9%)	340 (58.22%)		
Obesity					
Yes	161 (66%)	137 (46%)	298 (51%)	0.33	1.081
No	143 (50%)	143 (50%)	286 (49%)		
Chronic kidney disease					
Yes	19 (82.6%)	4 (17.4%)	23 (4%)	**0.003** ^*^	1.626
No	285 (50.8%)	276 (49.2%)	561 (96%)		
Chronic liver disease					
Yes	2 (40%)	3 (60%)	5 (0.9%)	0.588	0.766
No	302 (52.2%)	277 (47.8%)	579 (99.1%)		
Chronic lung disease (asthma, COPD, and/or TB)					
Yes	11 (50%)	11 (50%)	22 (3.8%)	0.844	0.958
No	293 (52.1%)	269 (47.9%)	562 (96.2%)		
Cancer					
Yes	3 (25%)	9 (75%)	12 (2.1%)	0.058	0.475
No	301 (52.6%)	271 (47.4%)	572 (97.9%)		
Autoimmune disease					
Yes	1 (33.3%)	2 (66.7%)	3 (0.6%)	0.515	0.638
No	303 (52.2%)	278 (47.8%)	581 (99.4%)		
*Vital signs at admission*					
Blood pressure, *n* (%)					
HT	262 (55.3%)	212 (44.7%)	474 (81.16%)	**0.002** ^*^	1.447
Not HT	42 (38.20%)	68 (61.80%)	110 (18.84%)		
Systole (mmHg)	132 (30)	124 (14)	584	**<0.001** ^*^	N/A
Diastole (mmHg)	80 (19)	78 (12)	584	**0.001** ^*^	N/A
Heart rate (beats/min)	90 (19)	90 (20)	584	0.371	N/A
Body temperature (°C)	36.50 (0.50)	36.40 (0.40)	584	0.072	N/A
Respiratory rate (breath/min)	22 (6)	22 (5)	584	**0.046** ^*^	N/A
Peripheral oxygen saturation (%)	97 (4)	97 (3)	584	0.085	N/A
BMI (kg/m^2^)	25.39 (6.01)	25.08 (5.54)	584	0.567	N/A
*Laboratory findings at admission*					
SGOT	40 (36)	43 (42)	584	0.980	N/A
SGPT	37 (32)	45.50 (48)	584	0.119	N/A
Ureum	35 (26)	26 (22)	584	**<0.001** ^*^	N/A
Creatinine	1 (0.50)	0.80 (0.30)	584	**<0.001** ^*^	N/A
GFR	78.32 (36.99)	95 (39)	584	**<0.001** ^*^	N/A
BUN	16.36 (12.15)	12.15 (10.28)	584	**<0.001** ^*^	N/A
Electrolyte: sodium	133 (6)	134 (5)	584	**0.011** ^*^	N/A
Electrolyte: potassium	4 (0.90)	3.90 (0.60)	584	0.184	N/A
Electrolyte: chloride	99 (7)	101 (6)	584	**0.002** ^*^	N/A
Hemoglobin	13.60 (2.30)	13.19 (2.12)	584	0.218	N/A
Platelet	240,000 (139000)	273,000 (163500)	584	**0.047** ^*^	N/A
Basophil (%)	0.10 (0.10)	0.10 (0.20)	584	0.239	N/A
Eosinophil (%)	0.00 (0.40)	0.10 (0.40)	584	0.402	N/A
Neutrophil (%)	78.50 (14.70)	79.15 (13.70)	584	0.405	N/A
Lymphocyte (%)	13.70 (12.30)	13.26 (11.40)	584	0.351	N/A
Monocyte (%)	7.10 (4.50)	7.08 (3.15)	584	0.580	N/A

BMI, body mass index; BUN, blood urea nitrogen; COPD, chronic obstructive pulmonary disease; CVD, cardiovascular disease; GFR, glomerular filtration rate; GI, gastrointestinal; HT, hypertension; IQR, interquartile range; N/A, not applicable; RR, relative risk; SD, standard deviation; SGOT, serum glutamic-oxaloacetic transaminase; SGPT, serum glutamate pyruvate transaminase; TB, tuberculosis.

a Calculated using the chi-square test or Mann–Whitney test, *p*-value less than 0.05 are considered statistically significant.

*p*-value less than 0.05 is considered statistically significant.

On the other hand, Table 2 analyzed the association between CVD and its comorbidities with five clinical outcomes in COVID-19 pneumonia patients. The most common CVD was hypertension (48.1%), followed by MI (10.6%), CAD (9.2%), CHF (6.8%), HHD (3.1%), arrhythmia (1.7%), and others (0.7%). The in-hospital mortality rate was 24%, and patients were hospitalized for a median of 12 days. MI was the only CVD that increased in-hospital mortality (RR 2.105, *p* = 0.001). It was also significantly increased in patients with diabetes mellitus (RR 1.475, *p* = 0.011) and chronic kidney disease (RR 2.079, *p* = 0.013). Meanwhile, prolonged hospital stay was associated with any CVD (RR 1.553, *p* < 0.001), hypertension (RR 1.511, *p* = 0.001), MI (RR 1.969, *p* < 0.001), CHF (RR 1.595, *p* = 0.037), diabetes mellitus (RR 1.359, *p* = 0.014), and cerebrovascular disease (RR 2.203, *p* = 0.001). Diabetes mellitus also increases the risk of ventilator use (RR 1.449, *p* = 0.027).

**Table 2:** Cardiovascular diseases, non-cardiovascular comorbidities, and correlation with clinical outcomes.

Variable	In-hospital mortality		ICU admission		Ventilator use		Earlier death		Prolonged hospital stay	
	RR	*p*-value^a^	RR	*p*-value^a^	RR	*p*-value^a^	RR	*p*-value^a^	RR	*p*-value^a^
CVD										
Any CVD	1.302	0.094	1.230	0.247	1.250	0.201	0.943	0.806	1.553	**<0.001** ^*^
Hypertension	1.245	0.164	1.172	0.383	1.186	0.339	1.069	0.755	1.511	**0.001** ^*^
Hypertensive heart disease	0.962	1	0.360	0.313	1.003	1	0	0.275	1.039	1
Coronary artery disease	1.357	0.234	1.159	0.695	0.917	0.908	0.868	0.712	1.294	0.251
Chronic heart failure	1.392	0.264	1.318	0.459	1.403	0.309	0.888	0.858	1.595	**0.037** ^*^
Myocardial injury	2.105	0.001^*^	1.406	0.203	1.553	0.069	0.87	0.575	1.969	**<0.001** ^*^
Arrythmia	2.128	0.065	1.475	0.433	1.645	0.393	0.658	0.389	1.567	0.378
Other comorbidities										
Diabetes mellitus	1.475	0.011^*^	1.348	0.084	1.449	0.027^*^	0.944	0.809	1.359	**0.014** ^*^
Obesity	0.933	0.707	1.340	0.092	1.056	0.816	0.772	0.091	0.886	0.359
Cerebrovascular disease	1.144	0.908	0.644	0.585	1.473	0.268	0.827	0.683	2.203	**0.001** ^*^
Chronic kidney disease	2.079	0.013^*^	1.406	0.402	0.912	1	1.066	1	1.536	0.227
Chronic liver disease	0	0.345	0	0.579	0	0.578	N/A	N/A	0.517	0.653
Lung diseases	0.751	0.694	0.192	0.068	0.582	0.418	0.829	1	0.861	0.831
Cancer	1.044	1	0.794	1	0.786	1	0.550	0.564	0.863	1
Autoimmune	0	1	1.466	0.541	1.451	0.545	N/A	N/A	0.865	1

CVD, cardiovascular disease; ICU, intensive care unit; N/A, not applicable; RR, risk ratio.

a Calculated using the chi-square test, *p*-value less than 0.05 are considered statistically significant.

*p*-value less than 0.05 is considered statistically significant.

## Discussion

This is a cohort study describing the cardiovascular prognosis of confirmed COVID-19 patients who presented at the Persahabatan Central General Hospital, Jakarta Islam Hospital Cempaka Putih, Yarsi Hospital, and Cengkareng District General Hospital during the study period. Among 2,569 patients, MI and hypertension were the most significant predictors of a higher risk of hospitalization and complications than other comorbidities.

The association between CVD and prolonged hospital stay demonstrated in this study aligns with a meta-analysis by Sabatino et al. (14), where pre-existing cardiovascular comorbidities had a significant association with in-hospital mortality and fatality rate. Another study that supported the finding is a systematic review and meta-analysis by Matsushita et al. (15), where hypertension, diabetes, and CVD were significantly associated risk factors for severe COVID-19, hence indicating a prolonged hospital stay due to the severity of COVID-19.

Our study shows that MI was significantly associated with in-hospital mortality and prolonged hospital stay. The findings are consistent with research by He et al. (16), In-hospital mortality was significantly higher in COVID-19 patients with myocardial injury (*p* = 0.013). It was also similarly claimed in a study by Arévalos et al. (17), where the myocardial injury was considered an independent predictor of in-hospital mortality (*p* < 0.001). Significantly worse clinical outcomes of the patients with myocardial injury might have caused the prolonged hospital stay.

The study demonstrated that hypertension was significantly associated with a prolonged hospital stay for COVID-19 patients. A systematic review and meta-analysis support the result by Ramphul et al. (18), which has stated that the odds ratio of a hypertensive patient with a severe outcome of COVID-19 was 2.58 (95% CI: 2.16–3.08, *p* < 0.01). According to another study by Hu et al. (19), patients with hypertension were more likely to develop ARDS and be admitted to ICU. Hypertension frequently occurs in the elderly and subjects with comorbidities, hence might prolong the length of stay due to the late recovery (20). Uncontrolled blood pressure may have exacerbated the clinical symptoms of COVID-19 patients with comorbidities by contributing to vascular remodeling and stiffness, endothelial dysfunction, and atherosclerosis (20).

This study also demonstrated a significant association between CHF and prolonged hospital stay in COVID-19 patients, consistent with a study by Standl et al. (21), with the finding that patients with a history of heart failure presented with lower oxygen saturation (*p* < 0.001), experienced longer lengths of hospital stay (*p* < 0.001), increased risk of mechanical ventilation (*p* < 0.001), and in-hospital mortality (*p* < 0.002).

For other non-cardiovascular comorbidities in this study, diabetes mellitus and chronic kidney disease were significantly associated with in-hospital mortality, and cerebrovascular disease was associated with a prolonged hospital stay. This finding was in line with previous research studied by Surendra et al. (22) in Jakarta, which stated that the risk of death among COVID-19 patients increased by hypertension, diabetes mellitus, and chronic kidney disease.

The evidence described above, accumulated in clinical practice, would contribute to a further understanding of the etiology mechanism of the diseases. Acute infections enhance a proinflammatory environment with more TNF-α produced, exaggerating the symptoms’ exacerbation due to the increased metabolic demand leading to acute decompensated heart failure (23). The combination between heart failure and septic shock led to an in-hospital mortality rate of 70%–90%, compared with 20% in septic patients without cardiovascular impairment (23, 24).

COVID-19 and CVD are linked to increased morbidity and in-hospital mortality. ACE-2 is upregulated in patients with cardiovascular diseases, increasing susceptibility to COVID-19 and risk of more severe clinical features. ACE-2 is postulated as a host cellular receptor, indicating the susceptibility of heart and blood vessels to a direct invasion of SARS-CoV-2 (24). In patients with cardiovascular diseases, the risk of developing acute decompensated heart failure is higher due to increased alveolar fluid and impaired pathogen clearance, resulting in worse clinical manifestations of pneumonia (25).

Exacerbating the inflammatory environment leads to a worse disease progression, with the risk of thrombosis activation due to inflammation as a potential etiology of myocardial injury in COVID-19 (26). The myocardial injury also leads to a more proinflammatory state, worsening the clinical manifestation of COVID-19 pneumonia (6, 26). Strong interferon-mediated responses could contribute to myocardial dysfunction (27). Hence clinical deterioration is associated with prolonged hospital stays and increased risk of death (27).

This study has some limitations. The first limitation is that not all selected study sites routinely performed troponin-level tests. Hence troponin level analysis was not included. Several other biomarkers that were not incorporated into this paper (hsCRP, hsTn and BNP/NT-proBNP) were also a limitation for study analysis. Although this does not reduce the power of this study, the analysis of these biomarkers on mortality rates can support various previous studies related to their pathophysiology and mortality in COVID-19 patients (28, 29). In addition, electrocardiography and echocardiography were not routinely performed in all sites, so the analysis of the results could not be included. The death-on-arrivals and death less than 24 h of admission cases were not analyzed due to inconclusive causes of death and incomplete laboratory results.

## Conclusion

COVID-19 pneumonia in patients with CVD, specifically MI and hypertension, worsens the COVID-19 clinical outcomes. Based on these findings, it is essential to perform comprehensive screening for cardiovascular comorbidities in COVID-19 pneumonia patients and proper medical treatment for them.

## Data availability statement

The original contributions presented in the study are included in the article/supplementary material, further inquiries can be directed to the corresponding author.

## Ethics statement

The studies involving human participants were reviewed and approved by Ethics Committee of Health Research of Persahabatan Hospital. The patients/participants provided their written informed consent to participate in this study.

## Author contributions

EB, FM, SA, YH, and NS: conceptualization and investigation. EB, FM, SA, and EW: methodology. FM and SA: software. EB, YH, and NS: validation. EB, FM, and SA: formal analysis. EB, CS, EI, and PA: resources. FM: data curation and visualization. FM and SA: writing—original draft preparation. EB, FM, SA, CS, EI, PA, YH, EW, and NS: writing—review and editing. EB and NS: supervision. SA: project administration. EB: funding acquisition. All authors contributed to the article and approved the submitted version.

## Conflict of interest

The authors declare that the research was conducted in the absence of any commercial or financial relationships that could be construed as a potential conflict of interest.

## Publisher’s note

All claims expressed in this article are solely those of the authors and do not necessarily represent those of their affiliated organizations, or those of the publisher, the editors and the reviewers. Any product that may be evaluated in this article, or claim that may be made by its manufacturer, is not guaranteed or endorsed by the publisher.
